# Probing the dielectric, piezoelectric and magnetic behavior of CoFe_2_O_4_/BNT-BT_0.08_ composite thin film fabricated by sol-gel and spin-coating methods

**DOI:** 10.1038/s41598-018-36232-3

**Published:** 2018-12-14

**Authors:** Marin Cernea, Bogdan Stefan Vasile, Vasile Adrian Surdu, Roxana Trusca, Cristina Bartha, Floriana Craciun, Carmen Galassi

**Affiliations:** 1 National Institute of Materials Physics, P.O. Box MG-7, Bucharest-Magurele, 077125 Romania; 2 University POLITEHNICA of Bucharest, 060042 Bucharest, Romania; 3 Istituto di Struttura della Materia-CNR (ISM-CNR), Area di Ricerca di Tor Vergata, I-, 00133 Roma, Italy; 4 CNR-ISTEC, Institute of Science and Technology for Ceramics, Via Granarolo 64, I-, 48018 Faenza, Italy

## Abstract

We investigated in this paper a novel bilayer composite obtained by sol-gel and spin coating of the ferroelectric 0.92Na_0.5_Bi_0.5_TiO_3_–0.08BaTiO_3_ (abbreviated as BNT-BT_0.08_) and ferromagnetic CoFe_2_O_4_ phases, for miniature low-frequency magnetic sensors and piezoelectric sensors. This heterostructure, deposited on Si-Pt substrate (Si-Pt/CoFe_2_O_4_/BNT-BT_0.08_), was characterized using selected method such as: X-ray diffraction, dielectric spectroscopy, piezoelectric force microscopy, SQUID magnetometry, atomic force microscopy/magnetic force microscopy, and advanced methods of transmission electron microscopy. CoFe_2_O_4_/BNT-BT_0.08_ ferromagnetic–piezoelectric thin films show good magnetization, dielectric constant and piezoelectric response. The results of analyses and measurements reveal that this heterostructure can have applications in high-performance magnetoelectric devices at room temperature.

## Introduction

In recent years, the manufacture of composite materials from components with different macroscopic properties was studied extensively^1–5^. These composites have applications in electronic devices with novel distinct functionalities^6–8^. Oxide heterostructure thin films with electric and magnetic properties were prepared by various techniques, such as: sol-gel^9^, pulsed laser deposition^10^, rf sputtering^11^, tape-casting method^12^, etc. There are several reports on composites with electrical, ferroelectric and ferromagnetic behaviors, for example: ferroelectric-ferromagnetic composites (BiFeO_3_-CoFe_2_O_4_^13^, nickel ferrite-PZT and manganite-PZT^8^, CoFe_2_O_4_-BaTiO_3_^14^) and ferromagnetic-piezoelectric oxide heterostructures (La_0.7_Sr_0.3_MnO_3_-PbZr_0.2_Ti_0.8_O_3_^15^, CoFe_2_O_4_–PZT^10^). There are few reports on lead free ferroelectric (Na_0.5_Bi_0.5_TiO_3_)–magnetostrictive (CoFe_2_O_4_) particulate composites^16–19^ but bilayer ferrite–piezoelectric composites of CoFe_2_O_4_ and BNT-BT_0.08_ have not been reported so far a heterostructure composed of a piezoelectric and a magnetic one.

In this work we have created a heterostructure thin film composed from piezoelectric and magnetic materials. We have investigated the composite thin film Si-Pt/CoFe_2_O_4_/BNT-BT_0.08_ in which BNT-BT_0.08_ and cobalt ferrite (CoFe_2_O_4_) films were deposited in two subsequent steps on Si-Pt buffer, by sol-gel and spin-coating techniques. Cobalt ferrite was chosen because is an important component for multiferroic heterostructure thin films or composites due to its high coercivity, moderate magnetization and highest magnetostriction coefficient^20^. (Bi_0.5_Na_0.5_)TiO_3_ (BNT) doped with BaTiO_3_ (BT) is selected as ferroelectric layer because BNT-BT_0.08_ is considered a good candidate to replace the lead-based piezoelectric materials^21,22^. It has been shown previously that the (1-x)BNT-xBT (BNT-BT_x_) solid solution has in the compositional domain x = 0.06–0.10 a nearly morphotropic phase boundary (MPB)^23,24^ where rhombohedral Bi_0.5_Na_0.5_TiO_3_ and tetragonal BaTiO_3_ phases coexist. Compared with BNT ceramic, the BNT-BT_0.08_ composition placed in the MPB provides improved poling and piezoelectric properties^23,25^.

## Results and Discussion

### Structural characterization of Si-Pt/CoFe_2_O_4_/BNT-BT_0.08_ bilayer heterostructure

**Figure 1:**
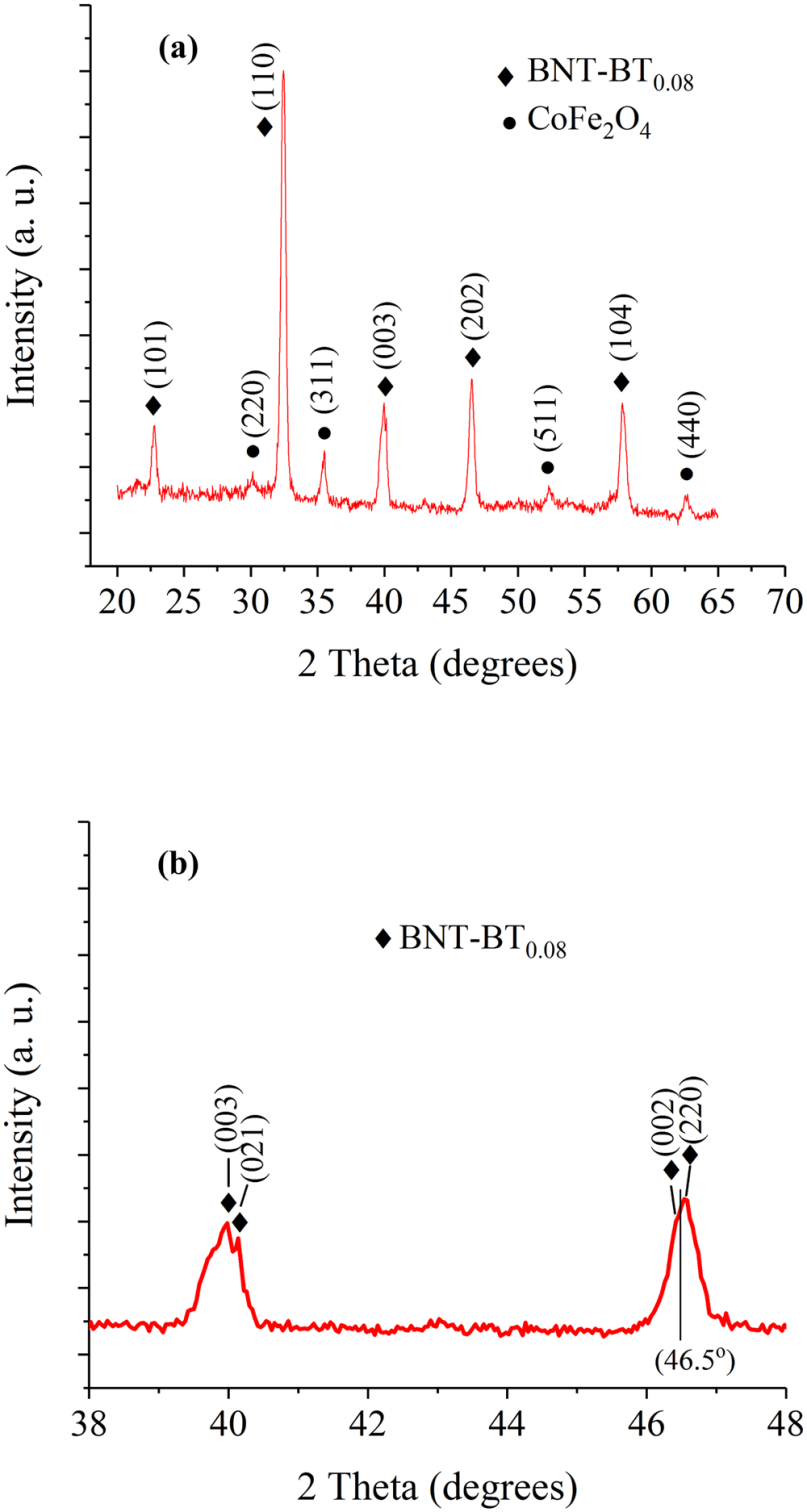
(**a**) XRD patterns of the Si-Pt/CoFe_2_O_4_/BNT-BT_0.08_ heterostructure thin film and (**b**) detail between 38° and 48°.

XRD spectra recorded in grazing incidence diffraction geometry of the Si-Pt/CoFe_2_O_4_/BNT-BT_0.08_ heterostructure thin film are shown in Fig. 1.

**Figure 2:**
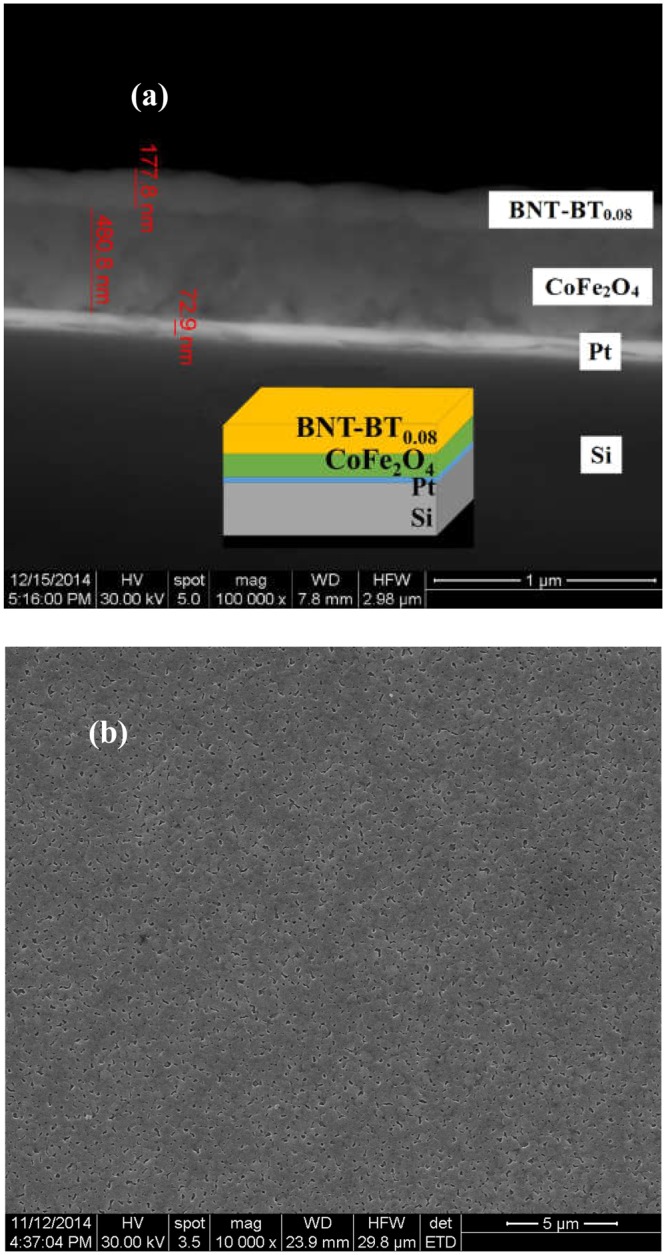
(**a**) Cross-section SEM image of Si-Pt/CoFe_2_O_4_/BNT-BT_0.08_ heterostructure thin film and a schematic representation inset; (**b**) SEM image of the composite film surface.

As expected, it can be observed that the sample is biphasic (CoFe_2_O_4_ and BNT-BT_0.08_). All the diffraction peaks could be indexed with a spinel structure (Fd3m space group) for CoFe_2_O_4_ phase (JCPDS: 04-005-7078)^26^ and a perovskite rhombohedral structure for Bi_0.5_Na_0.5_TiO_3_ rhombohedral phase (JCPDS: 04-015-0482)^27^, respectively.The rhombohedral phase of BNT-BT_0.08_ layer at room temperature, is characterized by (003)/(021) peak splitting between 39° and 41° and, a single (202) peak between 45° and 48°, as indicates the JCPDS: 04-015-0482^27^ while BNT-BT_0.08_ tetragonal phase shows splitting of the (200) and (220) peaks at 2*θ* ~ 46.5° (JCPDS: 04-011-3919)^28^. Figure 2(b) shows a not symmetrical peak at 45°, that can be deconvoluted in the (200) and (220) peaks, suggesting the existence of BNT-BT_0.08_ tetragonal phase. This result is in good agreement with the paper of Xu *et al*.^29^, which reported that the MPB of BNT-BT_x_ exists for 0.06 < *x* < 0.10.

Figure 2 shows the SEM images obtained by backscattering for Si-Pt/CoFe_2_O_4_/BNT-BT_0.08_ heterostructure thin film. The surface of BNT-BT_0.08_ top film of Si-Pt/CoFe_2_O_4_/BNT-BT_0.08_ heterostructure shows pores formed as a result of the removal the organic species during the calcination of the film gel. (Fig. 2(b)).

The cross-section and plan-view images of the heterostructure thin film indicate a granular structure, specific for the thin films prepared by the sol-gel method, with grain of polyhedral shape and various sizes. As can be seen on Fig. 2(a,b), some pores on the surface are connected while the internal pores are in small quantities and not connected. Therefore, the porosity of the surface is much higher than internal porosity. No intermediate layer is observed and the interface between CoFe_2_O_4_ and BNT-BT_0.08_ layers is clear, which suggests that both single-phase CoFe_2_O_4_ and BNT-BT_0.08_ films can coexist without interface diffusion. Thus results are agreement with the XRD patterns.

As it results from BSE/SEM image in Fig. 2(a), the thickness of BNT-BT_0.08_ layer is in the 150–180 nm range, while the thickness of CoFe_2_O_4_ bottom layer is about 480–490 nm.

**Figure 3:**
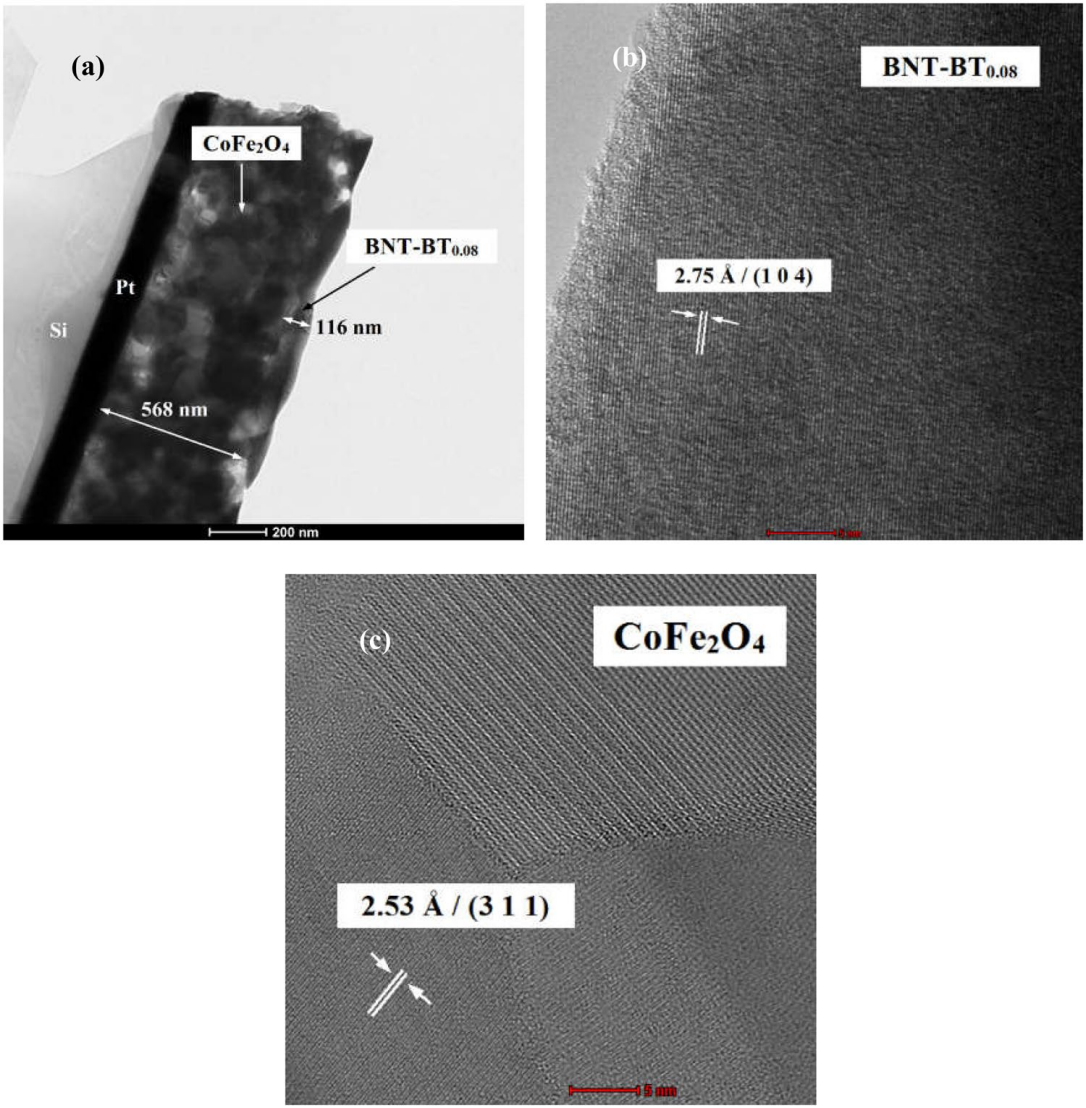
(**a**) TEM image of Si-Pt/CoFe_2_O_4_/BNT-BT_0.08_ heterostructure thin film, (**b**) HR-TEM image of BNT-BT_0.08_ layer and (**c**) HR-TEM image of CoFe_2_O_4_ layer.

Cross-sectional transmission electron microscopy shows BNT-BT_0.08_ top layer with thickness of 116 nm deposited on CoFe_2_O_4_ thin film of 568 nm thickness (Fig. 3(a)).

The high resolution TEM images (Fig. 3(b,c)) confirm the existence of CoFe_2_O_4_ cubic phase (*d* = 2.53 Å and (311) plane) and Na_0.5_Bi_0.5_TiO_3_ rhombohedral phase (*d* = 2.75 Å corresponding to the (104) crystallographic plane).

**Figure 4:**
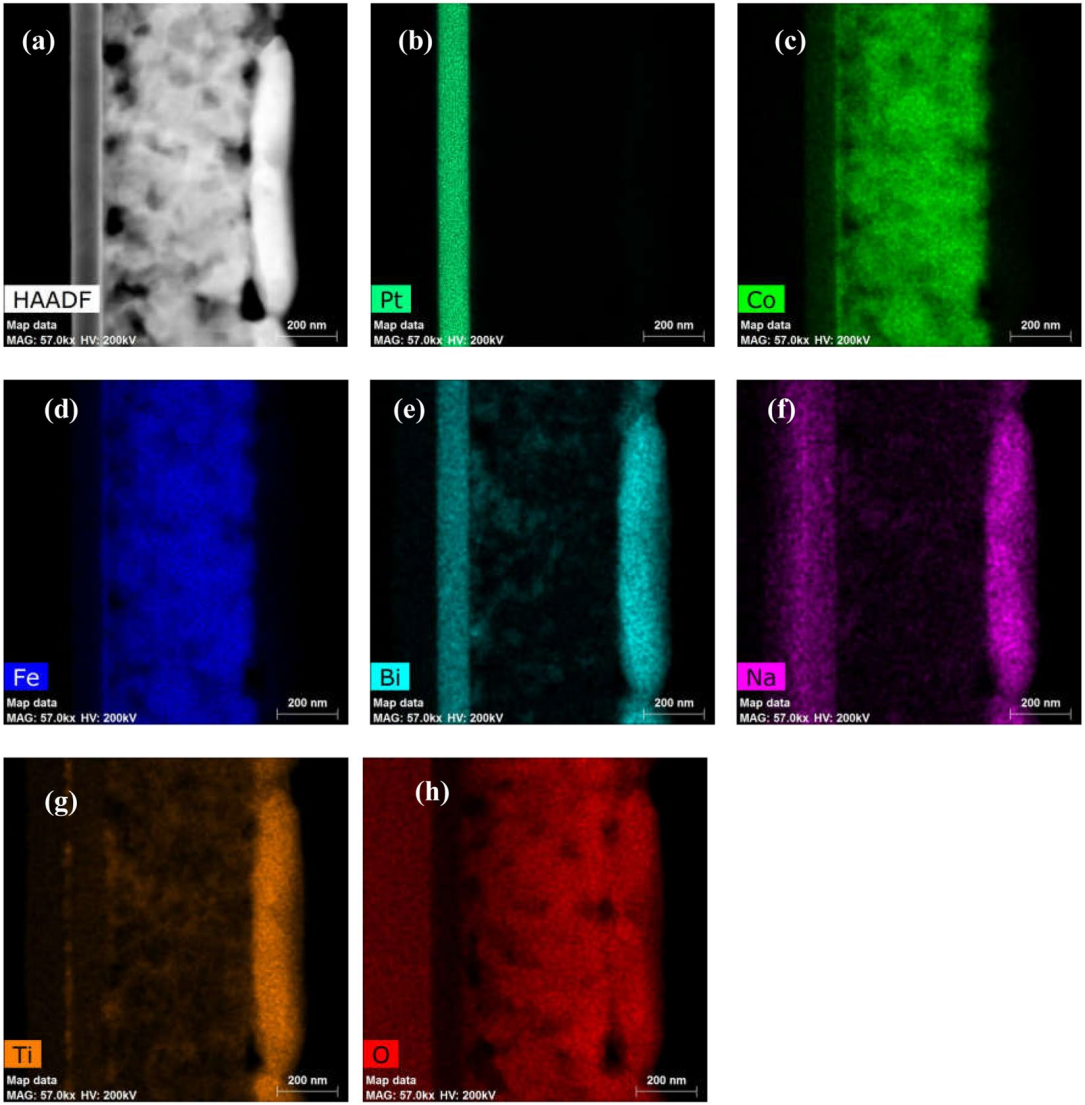
STEM of Si-Pt/CoFe_2_O_4_/BNT-BT_0.08_ heterostructure thin film. (**a**) High-angle annular dark-field imaging (HAADF). (**c**–**h**) Elemental maps of Co (**c**), Fe (**d**), Bi (**e**), Na (**f**), Ti (**g**) and O (**h**) for CoFe_2_O_4_/BNT-BT_0.08_ bilayered composite deposited on Pt (**b**) substrate.

High-angle annular dark-field (HAADF) imaging in scanning transmission electron microscopy (STEM) was also used in order to further confirm the bilayered structure of Si-Pt/CoFe_2_O_4_/BNT-BT_0.08_ composite (Fig. 4).

As can be seen in Fig. 4(a), the two chemically different layers (CoFe_2_O_4_ and BNT-BT_0.08_) are clearly distinguished from each other. Elemental mapping clearly shows the homogenous dispersion of Co, Fe, Bi, Na and Ti elements into their corresponding layers (Fig. 4(c–g)).

### Magnetic properties

**Figure 5:**
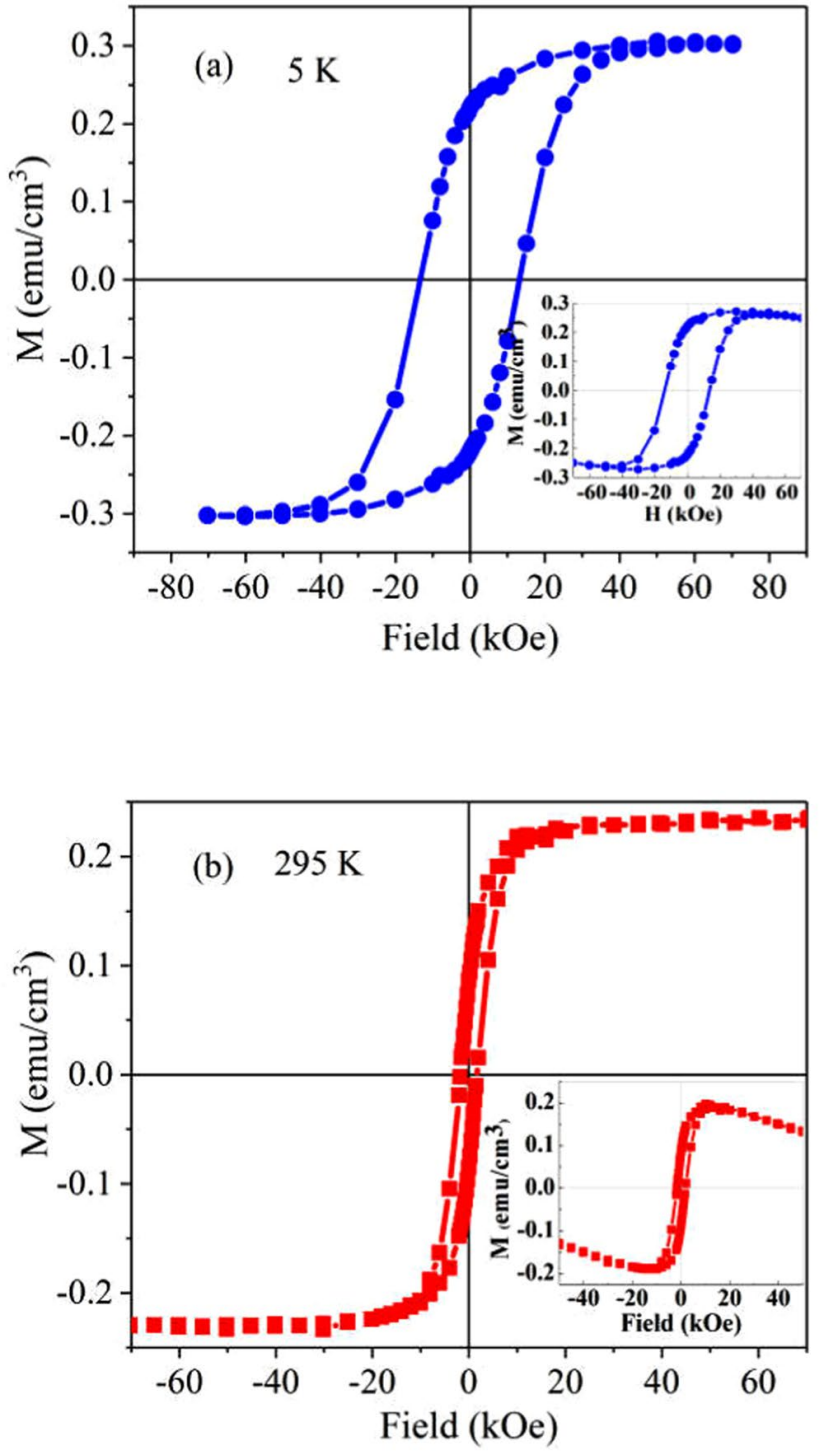
Magnetic hysteresis curves recorded at (**a**) 5 K and (**b**) 295 K for Si-Pt/CoFe_2_O_4_/BNT-BT_0.08_ layered composite.

The magnetic hysteresis loops of Si-Pt/CoFe_2_O_4_/BNT-BT_0.08_ bilayer heterostructure (Fig. 5(a,b)) were recorded at 5 K and 295 K, under a magnetic field of ≤ 70 kOe, perpendicular to the BNT-BT_0.08_ layer plane.

The composite CoFe_2_O_4_/BNT-BT_0.08_ exhibits magnetic hysteresis loops at 5 K and 295 K (Fig. 5(ab)), indicating an ordered magnetic structure. The hysteresis loops show a negative slope with value of −2·10^–3^ (emu/cm^3^)/Oe, due to the diamagnetic signal.

The values of remnant magnetization (*M*_*r*_), saturation magnetization (*M*_*s*_), and coercive magnetic field (*H*_*c*_) of the CoFe_2_O_4_/BNT-BT_0.08_ thin film heterostructure, after diamagnetic corrections, are listed in Table 1.

**Table 1:** Magnetic parameters of the CoFe_2_O_4_/BNT-BT_0.08_ bilayered heterostructure measured at 5 K and 295 K (results from the literature are reported as well, for comparison).

Heterostructure films samples	Temp. (K)	*Ms* (emu/cm^3^)	*M*_*r*_ (emu/ cm^3^)	*H*_*c*_ (kOe)
Si-Pt/CoFe_2_O_4_/BNT-BT_0.08_	5	0.3	0.22	13.63
	295	0.23	0.086	1.7
Pb(Zr_0.52_Ti_0.48_)O_3_/CoFe_2_O_4_/ SrTiO_3_ ^10^	RT	35.8	14.85	0.1
BNTKNNLTS-CZFMO^44^	RT	13.85	~ 0.50	7.5 × 10^–3^
Si-Pt/BNT-BT_0.08_/CoFe_2_O_4_^30^	5	0.136	0.08	15
	295	0.1	0.012	0.27

In Table 1: RT is the room temperature, SrTiO_3_ (100) single crystal is the substrate, BNTKNNLTS is (Bi_0.5_Na_0.5_TiO_3_)_0.97_(K_0.47_Na_0.47_Li_0.06_Nb_0.74_Sb_0.06_Ta_0.2_O_3_)_0.03_, and CZFMO is Co_0.6_Zn_0.4_Fe_1.7_Mn_0.3_O_4_.

As can see in Table 1, the magnetic parameters are higher for the CoFe_2_O_4_/BNT-BT_0.08_ composite thin film because the CoFe_2_O_4_ layer in this composite is thicker and has the crystallites larger than the CoFe_2_O_4_ layer in the BNT-BT_0.08_/CoFe_2_O_4_ composite structure^30^. These results are in good agreement with ref.^31^. In the CoFe_2_O_4_/BNT-BT_0.08_ bilayered heterostructure, the thickness of CoFe_2_O_4_ bottom layer is about 480–490 nm and crystallites size of ~65 nm while in the BNT-BT_0.08_/CoFe_2_O_4_ composite thin film, the thickness of CoFe_2_O_4_ layer is ~280 nm and crystallites size is ~20 nm^30^.

The low values of magnetization and coercive field, as well as the diamagnetic component of magnetization, indicate the behavior of a diluted magnetic oxide which may be due to Bi_0.5_Na_0.5_TiO_3_ and BaTiO_3_ (BNT**–**BT_0.08_) which are considered as diluted magnetic oxide^32^ and also, due of a very small contributions of the oxides from substrate. As we mentioned, the substrate is made of Si/SiO_2_(450 nm)/TiO_2_(15 nm)/Pt(100 nm); TiO_2_ being recognized as diluted magnetic oxide^33^, like also SiO_2_^34^.

The different component phases of the heterostructures, their crystallographic parameters, the thickness ratio between the magnetic and piezoelectric phases, the stress condition and the nature of the substrate are factors that reduce the magnetic parameters of our heterostructure compared with ferromagnetic–piezoelectric bilayer composites previously reported^10,33^. CoFe_2_O_4_ is a hard magnetic material with high coercivity and moderate magnetization. BNT-BT_0.08_ layer deposited on CoFe_2_O_4_ influences the magnetization of the heterostructure by the residual strain which appears at the CoFe_2_O_4_/BNT-BT_0.08_ interface, as has been reported for CoFe_2_O_4_-PZT bilayers^10,35^.

**Figure 6:**
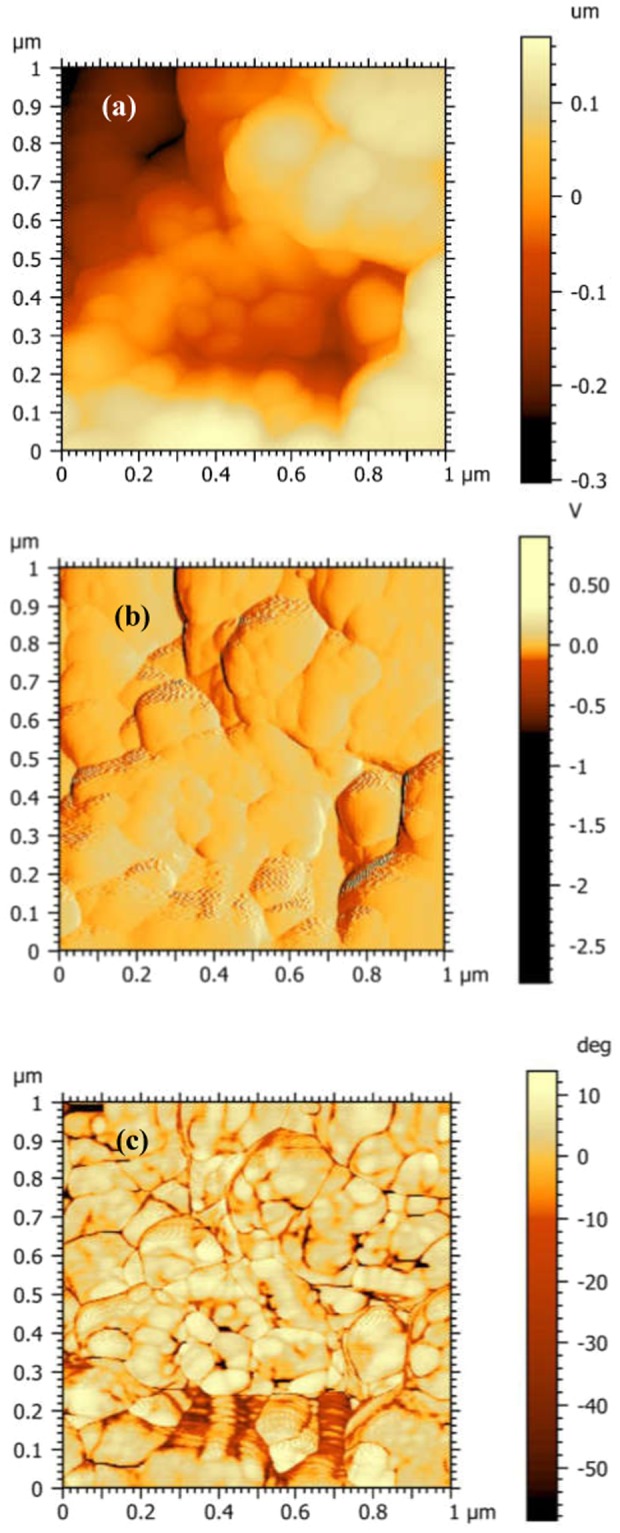
(**a**) AFM image and (**b**,**c**) MFM images: (**b**) amplitude and (**c**) phase for Si-Pt/CoFe_2_O_4_/BNT-BT_0.08_ layered heterostructure.

The bright and dark contrast of ferromagnetic domains indicates opposite direction of the magnetization, perpendicular to the surface of thin film. As can be seen in Fig. 6, Si-Pt/CoFe_2_O_4_/BNT-BT_0.08_ bilayered heterostructure shows magnetic domain at grains boundary and aggregates boundary region. The MFM images reveal magnetic properties for the composite film Si-Pt/CoFe_2_O_4_/BNT-BT_0.08_ and, a nanoscale magnetic domain configuration.

### Dielectric spectroscopy, tunability and leakage current measurements

**Figure 7:**
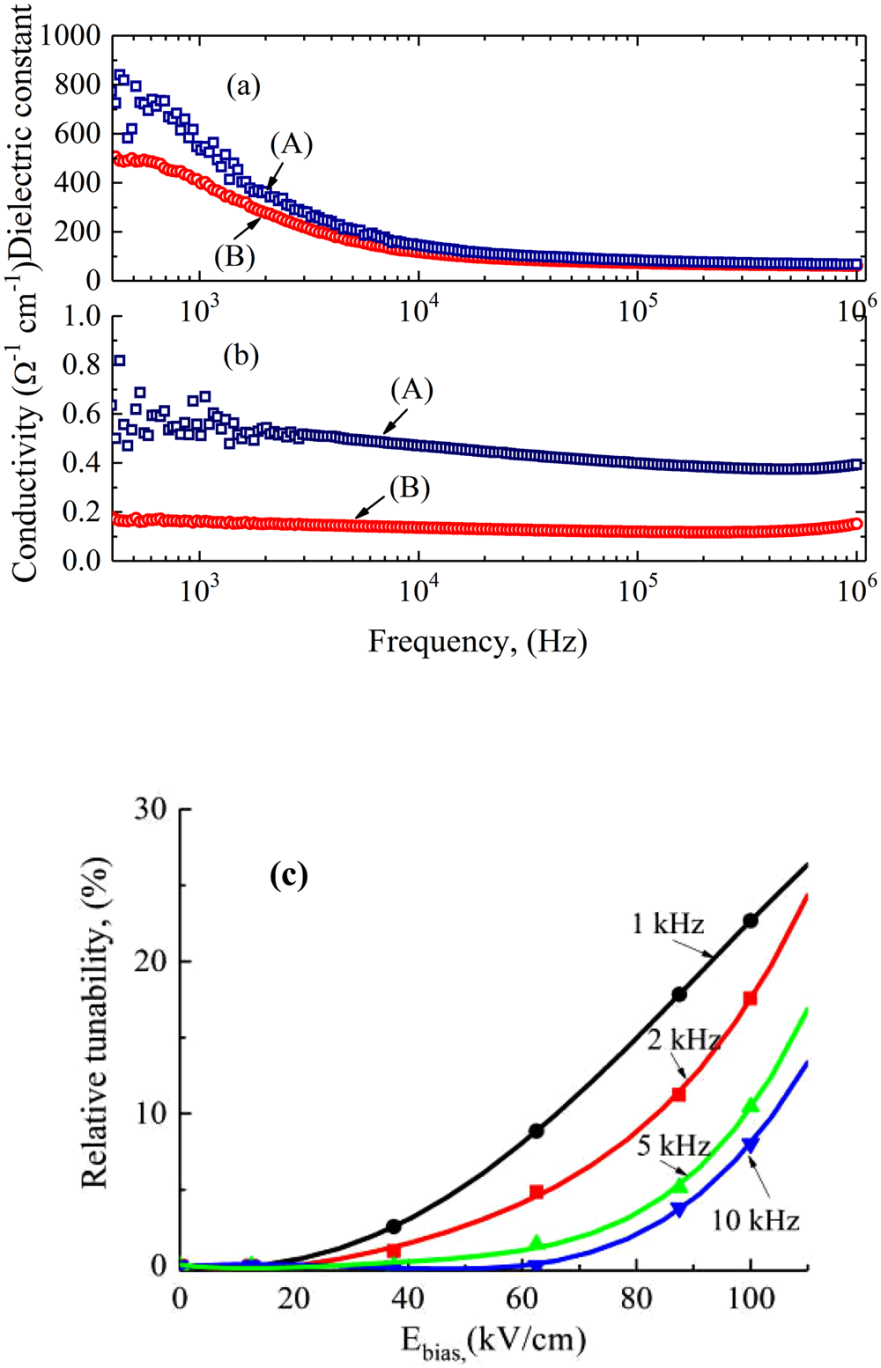
(**a**) Dielectric constant, (**b**) ac conductivity and (**c**) relative tunability for the Si-Pt/CoFe_2_O_4_/BNT-BT_0.08_ heterostructure thin films.

Figure 7 shows results of the dielectric spectroscopy measurements obtained for the bilayered heterostructure CoFe_2_O_4_/BNT-BT_0.08_ (Fig. 7(a,b)). The two different curves correspond to different capacitors (A and B). As can be seen in Fig. 7(a), the dielectric constant increases at low frequency and remains constant at higher frequency.

One must take into account the heterostructure type of the samples, with a layer of CoFe_2_O_4_ deposited over the ferroelectric layer BNT-BT_0.08_. The dielectric measurements are performed on the capacitor between the bottom electrode (Pt) and the top electrode (Au foil) applied over the CoFe_2_O_4_ layer. Therefore the measurements are carried out over a very small capacitance (CoFe_2_O_4_) in series with a high capacitance (BNT-BT_0.08_) which results in an equivalent capacitance smaller that of CoFe_2_O_4_ layer. This explains the small dielectric constant of the heterostructure.

CoFe_2_O_4_/BNT-BT_0.08_ structure shows high dielectric constant and high ac conductivity at low frequency (below 1 kHz) which indicates a strong contribution of relaxation processes associated with conductivity (due to oxygen vacancies and other defects) in this sample. Variation of the dielectric properties with frequency of the heterostructure thin film can be due to an extrinsic behavior resulting from the microstructure deficiency. The deviation from flat conductivity at low-frequency is also due to the interface between the layers and the electrodes.

In ref.^8^, it has been reported that with increasing CoFe_2_O_4_ film thickness, the frequency dependence of dielectric constant becomes gradually stronger, due to Maxwell-Wagner polarization mechanism^36,37^. In multiphase composites, Maxwell-Wagner polarization at the interface of ferroelectric-ferromagnetic phases would lead to strong dependence of dielectric constant especially at low frequency^38^.

Measurements of the dielectric permittivity of samples under a dc bias electric field E yield the tunability factor (*n*_*r*_), according to the formula:

1 $${n}_{r}=\frac{\varepsilon (0)-\varepsilon (E)}{\varepsilon (0)}$$

where ε(E) is the dielectric constant under bias and ε(0) is the dielectric constant without bias electric field. The results for the CoFe_2_O_4_/BNT-BT_0.08_ sample are shown in Fig. 7(c). It can be observed that the CoFe_2_O_4_/BNT-BT_0.08_ heterostructure displays a tunability of 23% at 1 kHz and 100 kV/cm. This value decreases strongly when the frequency increases. This decreasing is associated with the strong dependence of dielectric constant on frequency.

**Figure 8:**
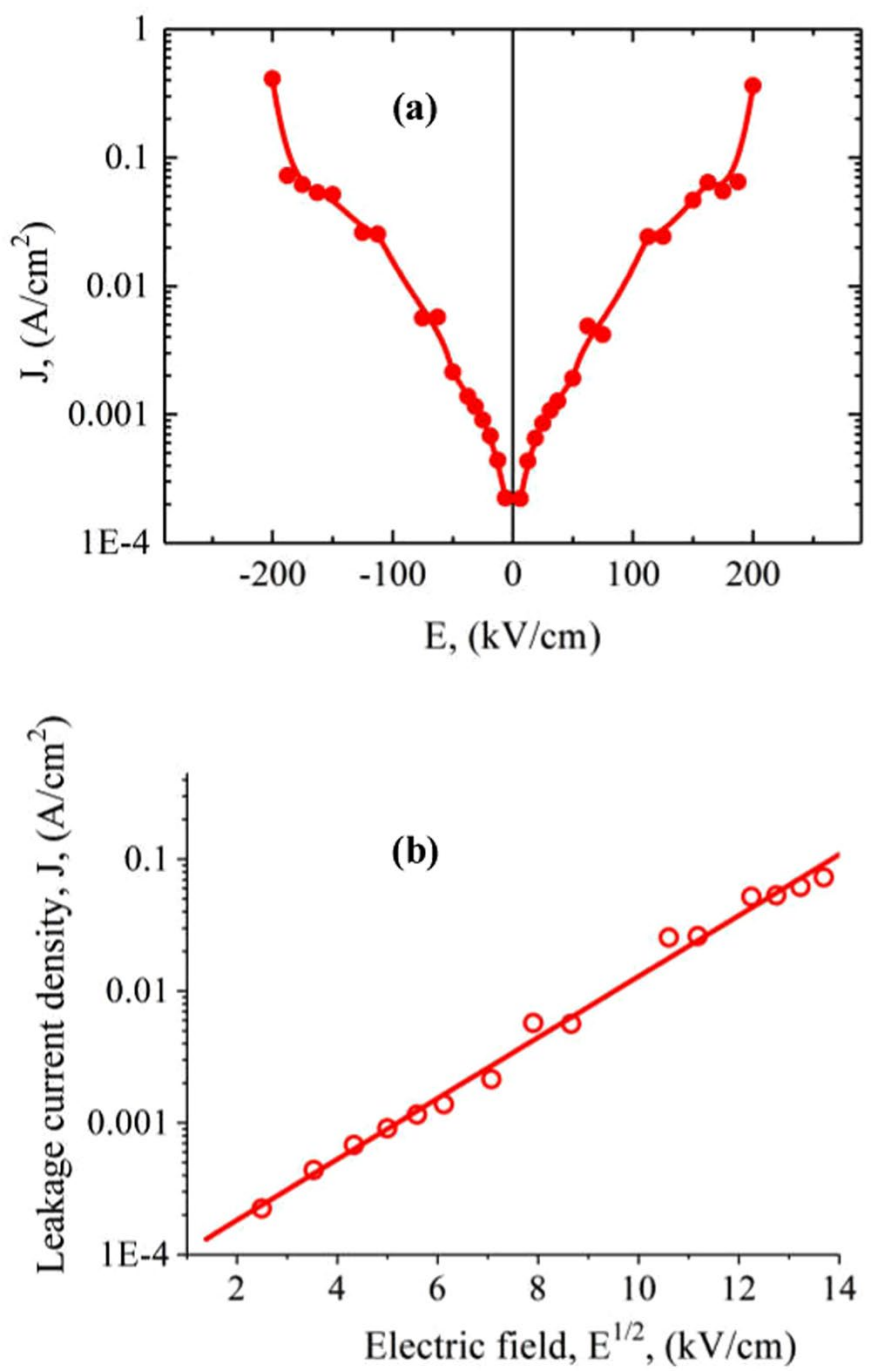
(**a**) Leakage current density (*J*) dependence on electric field E for the Si-Pt/CoFe_2_O_4_/BNT-BT_0.08_ heterostructure thin films; (**b**) leakage current density represented as a function of the square root (*E*^*1/2*^) of the field.

Leakage current density is a key factor for multiphase composite thick film and reflects the quality and reliability of a film. Therefore, it is important to investigate the leakage property and conduction mechanisms of the films. Leakage current measurements for the CoFe_2_O_4_/BNT-BT_0.08_ bilayer composite have been made at ambient temperature. The results are shown in Fig. 8(a,b).

It can be observed from Fig. 8(a) that the leakage current density shows symmetrical positive and negative arms. Leakage current density has the lowest value of about 1.5·10^–4^ A/cm^2^ at low electric field and shows a strong increase with electric field. The highest value of the current density measured on Si-Pt/CoFe_2_O_4_/BNT-BT_0.08_ heterostructure thin film, at an electric field of 200 kV/cm, was 4.0·10^–1^ A/cm^2^ (Fig. 8(a)). The leakage current is mainly attributed to conduction through grain boundaries in the BNT-BT_0.08_ layer^39^.

The parameters of the *J* (E) dependence law can give information about the possible conduction mechanisms in the composite structure. Thus a dependence of the type:

2 $$J\propto {E}^{2}$$

is characteristic for a space charge-limited conduction (SCLC) mechanism^40,41^. Alternatively, a dependence of the type:

3 $$\begin{array}{cc}J\propto {e}^{-\frac{1}{kT}({\rm{\Phi }}-a{E}^{1/2})} & 0\end{array}$$

is characteristic for a Schottky-type conduction mechanism^40,42^, related to the potential barrier created by the different Fermi levels of dielectric and metal. In equation (3), Φ is the height of the Schottky barrier height, *a* is a material constant and *k* is the Boltzmann constant^40,41^. Thus by plotting the obtained data as a function of E^2^ or E^1/2^ (on a semilogarithmic scale) it is helpful to discern among the different possible conduction mechanisms. For the CoFe_2_O_4_/BNT-BT_0.08_ heterostructure the plot of *J* vs *E*^2^ (not shown here) clearly showed that this law is not obeyed for any electric field range, therefore a SCLC mechanism was ruled out. Instead, as can be observed from Fig. 8(b), a plot of *J* (in log scale) vs *E*^*1/2*^ clearly shows that the leakage current is controlled by a Schottky barrier-type mechanism in almost all the field range.

It is concluded that the leakage mechanism found for the investigated hetereostructure points to the dominant role of the interfaces between the CoFe_2_O_4_ and NBT-BT_0.08_ and between these layers and the hybrid electrodes in the leakage current measurements.

### Piezoelectric properties

**Figure 9:**
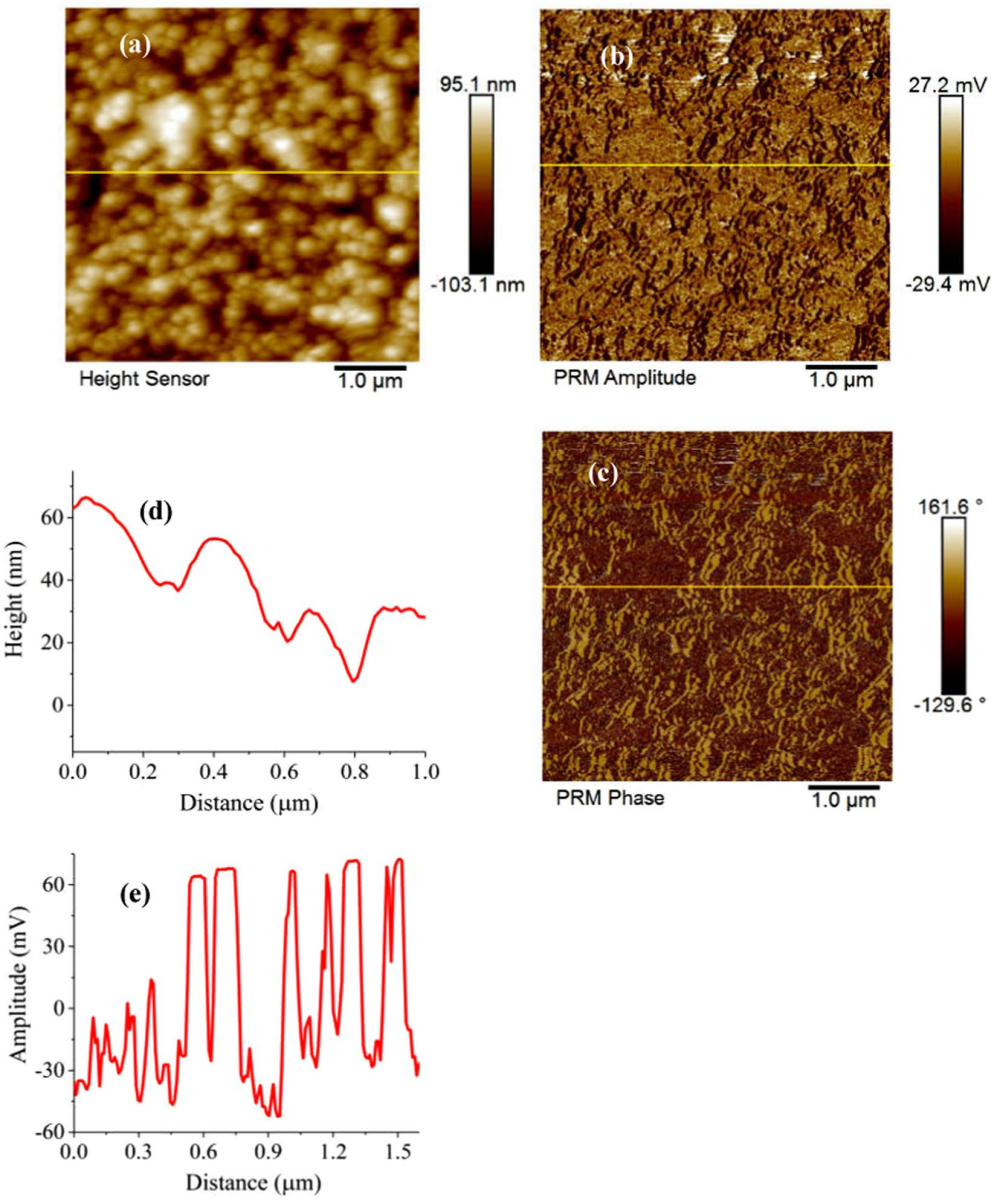
(**a**) AFM image, (**b**,**c**) PFM images: (**b**) amplitude and (**c**) phase, (**d**) line profile analysis of the morphology and (**e**) of the piezoresponse signal amplitude for the Si-Pt/CoFe_2_O_4_/BNT-BT_0.08_ heterostructure thin film.

**Figure 10:**
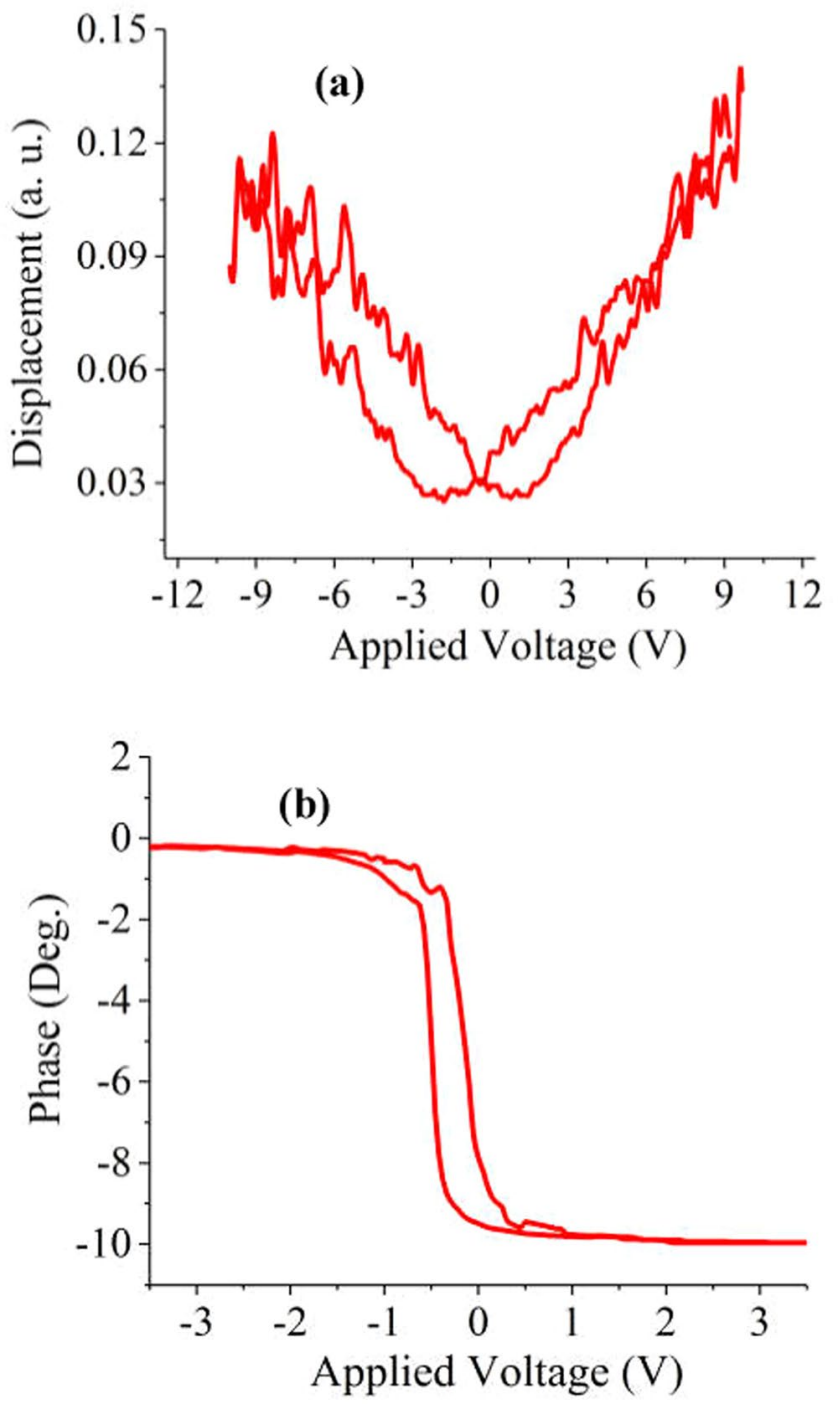
Local PFM hysteresis loops: (**a**) amplitude signal and (**b**) phase signal of Si-Pt/CoFe_2_O_4_/BNT-BT_0.08_ heterostructure thin film.

The ferroelectric behavior of the bilayered heterostructure CoFe_2_O_4_/BNT-BT_0.08_ was investigated by piezoelectric force microscopy (PFM) and their out-of-plane piezoelectric response is presented in Figs 9 and 10.

In the piezoelectric images, domains with opposite polarities exhibit different phase contrast. The CoFe_2_O_4_/BNT-BT_0.08_ composite thin film exhibits good piezoresponse over a large area (Fig. 9(b,c)). From the cross-section analysis, which simultaneously investigates the surface morphology (grain size) (Fig. 9(d)) and domain populations (domain size and response signal) (Fig. 9(e)), it can be seen that a majority of the domains, randomly distributed in the composite, have dimensions smaller than the grain size, suggesting a ferroelectric multidomains structure for the grains. Figure 10(a,b) reveal typical remnant piezoresponse amplitude and phase measurements, respectively, recorded as a function of applied dc voltage.

The polarization switching and hysteresis show clearly that the Si-Pt/CoFe_2_O_4_/BNT-BT_0.08_ bilayer thin film is ferroelectric at nanoscale level.

In summary, bilayer composite CoFe_2_O_4_/BNT-BT_0.08_ was deposited on Si-Pt substrate by spin-coating technique. This heterostructure thin film presented CoFe_2_O_4_ cubic phase and BNT-BT_0.08_ phase crystallized on the lattices of rhombohedral and tetragonal Bi_0.5_Na_0.5_TiO_3_. The composite CoFe_2_O_4_/BNT-BT_0.08_ film shows ferromagnetic, ferroelectric and piezoelectric properties. The values of magnetic parameters for CoFe_2_O_4_/BNT-BT_0.08_ heterostructure, with nanoscaled magnetic domain, were: *M*_*s*_ = 0.23 emu/cm^3^, *M*_*r*_ = 0.086 emu/cm^3^ and *H*_*c*_ = 1.7 kOe, at 295 K. CoFe_2_O_4_/BNT-BT_0.08_ structure shows high dielectric constant *ɛ*_*r*_ = 548 at 1 kHz and room temperature and good tunability of 23% at 1 kHz. PFM investigations suggested a ferroelectric multidomains structure for the grains of this composite thin film. The ratio of magnetic and electric properties of this composite depends on the thickness ratio of magnetic and piezoelectric layers. These results demonstrate the possibility of manufacturing 2-D materials for sensors with multiple functionalities (ferromagnetic, ferroelectric and piezoelectric).

## Methods

Si-Pt/CoFe_2_O_4_/BNT-BT_0.08_ ferromagnetic–piezoelectric bilayer composite was prepared on Si-Pt substrates, as follows. In the first step, 10 layers of CoFe_2_O_4_ were deposited on Si-Pt by spin-coating technique. The as-obtained thin film was calcined at 700 °C for 1 h, in air, in order to crystallize CoFe_2_O_4_. In the second step, 10 layers of BNT-BT_0.08_ sol-precursor were deposited by spin-coating on the cobalt ferrite thin film prepared earlier. The new structure was calcined at 800 °C for 5 min, in oxygen to crystallize the layer of BNT-BT_0.08_. The precursor sols of CoFe_2_O_4_ and BNT-BT_0.08_ were prepared by sol-gel technique as described in our previous works^42,43^, respectively. According to these reports, CoFe_2_O_4_ precursor sol was prepared from cobalt acetate (Co(CH_3_CO_2_)_2_·4H_2_O, 99.995%, Sigma-Aldrich), iron nitrate (Fe(NO_3_)_3_·9H_2_O, 99.99%, Sigma-Aldrich), citric acid (C_6_H_8_O_7_, 99%, Sigma-Aldrich), ethanol, acetic acid and distilled water. The precursor sol of BNT-BT_0.08_ was prepared from bismuth (III) acetate ((CH_3_COO)_3_Bi, 99.99%, Sigma-Aldrich), sodium acetate (CH_3_COONa, 99.995%, Sigma-Aldrich), barium acetate ((CH_3_COO)_2_Ba, 99%, Sigma-Aldrich), titanium (IV) isopropoxide (Ti{OCH(CH_3_)_2_}_4_) in isopropanol (Sigma-Aldrich), acetic acid, acetylacetone and formamide. In order to prepare CoFe_2_O_4_ precursor sol, the iron nitrate was dissolved in ethanol and separately, the amount of citric acid was dissolved in ethanol, at room temperature. The cobalt acetate was dissolved in a mixture of ethanol, acetic acid and water, at room temperature. The citric acid solution was added to the mixture solutions of Fe and Co, in a molar ratio of Co:Fe:citric of 1:2:3, and a cobalt ferrite precursor sol was obtained. The as obtained sol was used for deposition of CoFe_2_O_4_ thin film on Si-Pt substrate.

BNT-BT_0.08_ precursor gel was prepared using the following strategy: first, was prepared a mixture of sodium, bismuth and barium acetic solutions and separately, a mixed solution of titanium isopropoxide: isopropanol: acetylacetone in the volume ration 1:2:0.5. The complex solution of Na, Bi and Ba was added to the titanium isopropoxide solution under continuous magnetic stirring and heating at 85 °C, and a sol precursor of BNT-BT_0.08_ was prepared. Acetylacetone is used as stabilizer for the sol while formamide (N, N-dimethylformamide, Aldrich) was used in order to to avoid formation of the cracks in BNT-BT_0.08_ gel film during the thermal treatments of drying and crystallization. The films of CoFe_2_O_4_ and BNT-BT_0.08_ were deposited by spin casting at 3000 rpm for 20 seconds. After spin coating, the thin films were heated at 200 °C for 2 min to evaporate the solvent, and then at 400 °C for 4 min in air to eliminate organic components. The films were prepared by repeating (10 times) the deposition and pyrolysis cycle. The coated films of CoFe_2_O_4_ and BNT-BT_0.08_ were annealed at 700 °C for 1 h, in air, in order to crystallize CoFe_2_O_4_ and at 800 °C for 5 min, in oxygen to crystallize the layer of BNT-BT_0.08_. Thus, the final product is a Si-Pt/CoFe_2_O_4_/BNT-BT_0.08_ heterostructure thin film.

The structure of the heterostructure thin film was investigated by using a Bruker-AXS tip D8 ADVANCE diffractometer while the microstructure was investigated with a FEI QUANTA INSPECT F scanning electron microscope (SEM) with field emission gun and a Tecnai^TM^ G^2^ F30 S-TWIN transmission electron microscope (TEM) with a line-resolution of 1 Å, in high resolution transmission electron microscopy (HRTEM) mode. The crystalline structure of the samples was investigated also the selected area electron diffraction (SAED) method. A Titan Themis 200, transmission and scanning transmission electron microscope (S/TEM) was used for analysis of the chemical elements present in the samples. The magnetic hysteresis loops of the bilayer composite were recorded using a Quantum Design MPMS-5S SQUID magnetometer, in the temperature range of 5–355 K and an applied magnetic field of ≤3580 kA/m. The out-of-plane magnetic characteristics of the samples were observed with an atomic force microscope/magnetic force microscope (AFM/MFM), Model 5400, from Agilent Technology. Measurements of the capacitance and dielectric loss have been carried out with an HP 4194 A impedance bridge under an electric bias field with variable amplitude. A Radiant Technologies RT-66A system has been employed for measurements of leakage currents on the same heterostructure samples. PFM measurement of local piezoresponse was performed using a sequence of DC switching bias from −3 V to +3 V, superimposed on a AC modulation bias with a frequency of 14 kHz, using a PFM tip directly on the heterostructure surface.
